# The Addition of Diacutaneous Fibrolysis to a Pharmacological Intervention in Patients with Tension-Type Headache: A Randomized Controlled Trial

**DOI:** 10.3390/jcm11226716

**Published:** 2022-11-13

**Authors:** Sara Cabanillas-Barea, Luis Ceballos-Laita, Silvia Pérez-Guillén, Sandra Jiménez-del-Barrio, Pilar Pardos-Aguilella, Pere Ramón Rodríguez-Rubio, Andoni Carrasco-Uribarren

**Affiliations:** 1 Faculty of Medicine and Health Sciences, Universitat International de Catalunya, 08195 Barcelona, Spain; 2 Faculty of Health Sciences, Universidad de Zaragoza, 50009 Zaragoza, Spain; 3 Department of Surgery, Ophthalmology, Otorhinolaryngology and Physiotherapy, University of Valladolid, Calle Universidad S/N, 42004 Soria, Spain; 4 Clinical Research in Health Sciences Group, University of Valladolid, Calle Universidad S/N, 42004 Soria, Spain

**Keywords:** tension-type headache, instrumental treatment, quality of life, patient-reported outcome measures

## Abstract

Background: Tension-type headache (TTH) is the most common headache worldwide. Pharmacological interventions are the most investigated therapies in patients with TTH. The addition of physical therapy treatments such as diacutaneous fibrolysis (DF) may have promising results. The aim of this study was to investigate the addition of three sessions of DF to a pharmacological intervention in patients with TTH. Methods: A single-blinded randomized controlled trial was carried out. Participants were randomly assigned to the standard care group or to the DF group. Both groups received a pharmacological intervention. Three sessions of DF in the thoracic and cervicocranial region were added in the DF group. The impact caused by headache (HIT-6), headache intensity, and cervical range of motion were measured by blinded assessors at baseline, after the intervention, and at 1 month follow-up. Results: Eighty-two patients with TTH were included (41 standard care group; 41 DF group). Statistically significant differences were found between both groups in all the variables after the intervention and at 1 month follow-up (*p* < 0.001). No adverse effects or side-effects were reported during the study. Conclusions: The addition of three sessions of DF to a pharmacological therapy provided improvements in the impact caused by headache, headache intensity, and cervical range of motion after the intervention and at 1 month follow-up compared to a pharmacological therapy in isolation. Further research is needed to investigate long-term effects.

## 1. Introduction

According to the Global Burden of Disease (GBD), headache disorders are one of the global public health concerns [1]. Every day, 15.8% of the world’s population experiences headaches [2]. Headaches can be divided into primary and secondary. Among the primary, tension-type headache (TTH) is the most frequent [3]. This situation makes TTH a pathology with a tremendous social and economic impact [4].

The most common symptoms are bilateral pain in frontal and occipital regions, dull pain across the forehead, sides, or back of the head, and tenderness on the scalp or muscles of the neck, upper back, shoulders, and jaw [5,6]. To explain this symptomatology, peripheral sensitization of the tissues near the orofacial region is suggested [7,8].

Proposed treatments for TTH include pharmacological and nonpharmacological approaches [9]. Acetaminophen and nonsteroidal anti-inflammatory drugs (NSAIDs) are among the most used pharmacological treatments according to the recommendations of the main clinical guidelines [10]. The European Federation of Neurological Societies intends to promote nonpharmacological treatments for this pathology, which have fewer side-effects than pharmacological ones [11]. However, a definitive treatment has not been described, nor has a treatment dose been proposed for this condition. On the other hand, it has been observed that manual therapy, specifically the soft-tissue approach, reduces pain and improves the quality of life in patients with frequent episodic and chronic TTH [12].

Diacutaneous fibrolysis (DF) is an instrumental technique that allows approaching soft tissue. The systematic review and meta-analysis conducted by Cadellans-Arróniz et al. [13] showed that this instrumental technique is effective in improving symptoms and function in other musculoskeletal disorders such as lateral epicondylalgia [14], subacromial impingement syndrome [15,16], and patellofemoral pain [17,18]. Furthermore, it has been shown that this technique can be effective even in those clinical subgroups where peripheral sensitization may be present such as carpal tunnel syndrome [19,20].

Considering that DF was shown to be effective in treating the abovementioned neuromuscular conditions [13], and taking into consideration the lack of studies investigating the efficacy of DF in patients with TTH, the aim of this study was to investigate the effects of adding DF to a standard care based on pharmacological therapy in the frequency of headache, impact of the headache, headache intensity, cervical mobility, and patient satisfaction in patients with TTH.

## 2. Materials and Methods

### 2.1. Study Design

A randomized controlled trial (simple 1:1) with two groups and one blinded evaluator was conducted. The protocol was registered in Clinicaltrials.gov (accessed on 23 October 2022) (NCT03056131). The study was carried out following the Declaration of Helsinki on Human Rights. It was evaluated and accepted by the local ethics committee (CEICA Number: PI15/0229). The subjects were evaluated and treated at the University of Zaragoza. This study followed the CONSORT criteria.

### 2.2. Selection Criteria

All patients were referred to the study of the Delicias sur Primary Care Center located in Zaragoza between October 2015 and May 2018. 

The inclusion criteria were age >18 years and diagnosis of frequent episodic or chronic TTH according to the criteria of the International Classification of Headache Disorders (ICHD) performed by the general practitioner: for frequent episodic TTH, at least 10 episodes of headache occurring on 1–14 days per month on average for >3 months; for chronic TTH, a headache occurring on ≥15 days per month on average for >3 months; for both types of TTH of headache bilateral location, pressing/tightening quality, mild or moderate intensity, not aggravated by routine physical activity, no nausea or vomiting (frequent episodic), and no more than one of photophobia, phonophobia, or mild nausea (chronic) [3]. The exclusion criteria were presenting skin damage, skin lesions, or vascular anomalies in the cranial–cervical area, concomitant treatment with platelet antiaggregant, previous cervical or cranial surgery, and patients with litigation or pending lawsuits.

### 2.3. Sample Size Calculation

The sample size was calculated on the basis of the frequency of days with a headache result and the HIT-6. The GRANMO 7.12 program with an α risk of 0.05, a bilateral test, and a β risk of 0.20 was used. For headache frequency, an estimated common standard deviation of 5 and a minimum difference to detect 3 days were used [21]. For HIT-6, an estimated common standard deviation of 5.4 points and a minimum difference to detect of 8 units were used [21,22]. The highest value obtained was chosen, which in this case corresponded to the frequency of headache. The result was 41 subjects per group.

Randomization of the participants (standard care *n* = 41; DF group *n* = 41) was performed by a statistician using Microsoft Excel 2010. A physical therapist reviewed the selection criteria for each participant, provided them with the necessary information, and asked them to sign an informed consent form if they agreed. Another researcher made the assessments throughout the study at baseline (T0), post intervention (T1), and at 1 month follow-up (T2). This researcher was blinded to the assignment group of each subject during all the study. After the first evaluation, this last researcher gave each subject a sealed and opaque envelope in which the statistician had previously included the subject‘s number and the assigned group. The physiotherapist who applied the treatment after opening the envelope was the only one who knew the group to which each participant belonged.

### 2.4. Outcome Measurements

**Figure 1:**
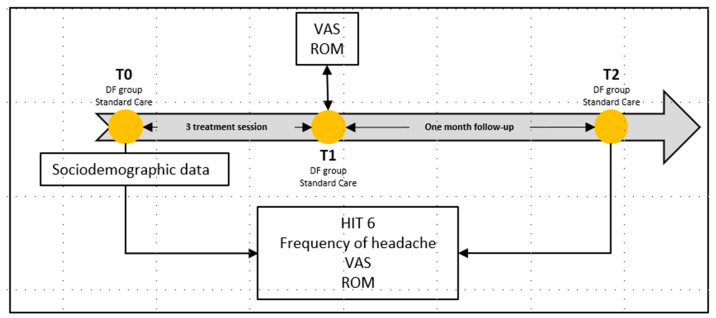
T0, baseline; T1, post intervention; T2, 1 month follow-up; DF, diacutaneous fibrolysis; VAS, visual analogue scale; ROM, range of movement; HIT-6, headache impact test-6.

The primary outcomes of this study were headache frequency and HIT-6, and the secondary outcomes were a cervical range of motion (ROM) per plane and headache intensity measured using a visual analogue scale (VAS). The frequency of headache episodes and HIT-6 were recorded at T0 and at T2. Nevertheless, the ROM and the VAS were recorded at three different moments: T0, T1, and T2 (Figure 1).

The number of days with headache during the last month was recorded for the frequency of headache episodes. 

The HIT-6 questionnaire measures the impact that headache has in the subjects [23]. This questionnaire consists of six items with four response options: never, six points; rarely, eight points; sometimes, 10 points; very often, 11 points; always, 13 points; the total score ranges from 36 to 78 points. The test–retest reliability is excellent (intraclass correlation coefficient (ICC) = 0.78 to 0.90), and the internal consistency is good (Cronbach’s alpha 0.89) [24].

The VAS is a tool that has been shown to be valid and reliable for measuring headache intensity [25]. A 10 cm vertical line was used; at one end of the line, the descriptor “no pain” was placed, with “the worst pain imaginable” at the opposite end, and patients were asked about the headache intensity at the time of assessment.

The ROM was assessed through the CROM device (floating compass; Plastimo Airguide, Inc, The Buffalo Groove, IL, USA), which was shown to have excellent test–retest and inter-examiner reliability (ICC > 0.80) [26]. The movement was recorded with the patient sitting with the back resting in the chair. For this study, the movement of each plane, sagittal, frontal, and transverse, was considered. 

At T2, the subjective self-perceived improvement was assessed. A seven-point Likert-type scale was used to record this question, with 0 being very much worse, 1 being much worse, 2 being slightly worse, 3 being the same, 4 being slightly better, 5 being much better, and 6 being very much better [21,23]. 

### 2.5. Interventions

Both groups received the standard care intervention based on pharmacological therapy. The treatment was prescribed by the primary care physicians of the Delicias sur Primary Care Center; acetaminophen and NSAIDs were mainly prescribed following the clinical guidelines recommendations [10].

The DF group received three interventions on alternate days. The treatment lasted 30 min, with 2 days separation between sessions.

**Figure 2:**
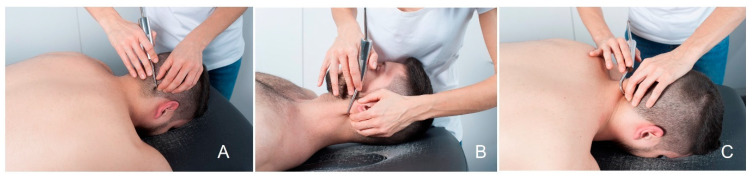
DF technique: (**A**) occipital base; (**B**) sternocleidomastoid muscle; (**C**) trapezius muscle.

Instrumental treatment was performed using the DF technique. A physiotherapist with 10 years of experience in manual therapy and instrumental treatment performed the intervention, who was blinded to outcome measurements. These procedures were performed with the hook fixed on the skin and underlying soft tissues (Figure 2). The position of the patient was in prone with the cervical spine neutral, placed into the facial hole and in supine position with cervical support to allow access to the muscular tissue. The DF was applied as deeply as possible following the intermuscular septum between the trapezius, levator scapulae, splenius cervicis, splenius capitis, and sternocleidomastoid muscles. In addition, scraping of the bony edges of the dorsal spinous processes, scapula, and occipital base was performed. The DF intervention was applied to start in the thoracic region, continuing through the scapular region, and ending in the cervical and cranial region. The efficacy of this type of intervention protocol has already been demonstrated in other dysfunctions such as carpal tunnel syndrome or anterior knee pain syndrome [17,19,20]. In these studies, three to five treatment sessions of 20 to 30 min duration were applied with 2 days separation between sessions [17,19,20]

International standards were followed to promote safety in the intervention, such as ruling out vascular diseases to reduce the risk of adverse events before the intervention [27]. Furthermore, if subjects felt worse during the study, they could withdraw, and efforts would be made to find the best way to help them.

### 2.6. Statistical Analysis

The SPSS 20.0 software (IBM, Armonk, Ney York, NY, USA) was used to conduct the statistical analysis. The mean and standard deviation were calculated for each dependent variable. The Kolmogorov–Smirnov test was used to determine the normal or non-normal distribution of the quantitative data. Sociodemographic and clinical data were compared between groups at baseline using a one-factor ANOVA or Mann–Whitney U test following the normally or non-normally distributed data. The chi-square test was used for the gender variable.

The between-group differences were analyzed using repeated-measures analysis of variance (ANOVA). The within-group differences were analyzed using the Student’s *t*-test. The significance level was set at *p* < 0.01. In addition, the effect sizes were calculated using Cohen’s d coefficient. An effect size <0.2 was considered small, of 0.5 was considered intermediate, and >0.8 was considered large [28].

## 3. Results

**Figure 3:**
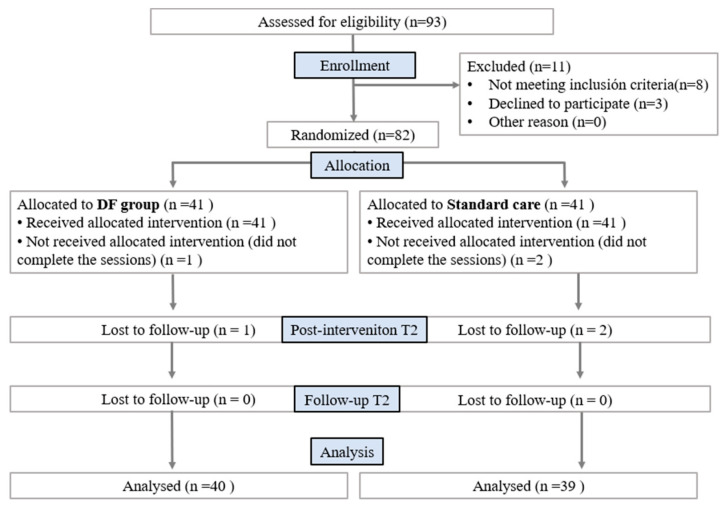
CONSORT flow diagram.

Between October 2015 and May 2018, 93 subjects were recruited, of whom 82 met the selection criteria. Three subjects did not complete the study (two did not complete the intervention protocol, and one did not attend the evaluation session). The analysis was completed with 79 participants (57 women and 22 men, 38.35 years ± 15.78) (Figure 3). Specifically, 14% of the sample had arterial hypertension, 9% had a digestive pathology, 5% had a respiratory pathology, and 4% had heart disease. The sociodemographic and clinical data at T0 are described in Table 1. No statistically significant differences were found for any variable at T0 (*p* > 0.05). 

### 3.1. Self-Perceived Impact Caused by the Headache, HIT-6, and Frequency of Headaches

A significant group by time interaction was found for HIT-6 (F = 27.26; *p* < 0.001) and for the frequency of headache (F = 29.10; *p* < 0.001) at T2. The DF group achieved higher changes than the standard care in HIT-6 (Δ6.66 (2.16 to 11.15) and in frequency of headaches (Δ9.66 (3.85 to 15.47). Table 2 provides T0 and T2 session data, within-group and between-groups differences, and effect sizes.

### 3.2. Intensity of Headache, VAS

A repeated-measures ANOVA showed a significant group by time interaction at T1 for headache intensity (F = 11.28; *p* = 0.001). The DF group showed a greater decrease in headache intensity than the standard care (Δ1.77; 0.56 to 2.98). The between-group difference was not maintained at T2 (F = 2.36; *p* = 0.129) (Table 3). 

### 3.3. Range of Motion 

The ANOVA analysis showed a significant group by time interaction for sagittal (F = 39.61; *p* < 0.001), frontal (F = 46.87; *p* < 0.001), and transversal ROM (F = 27.33; *p* < 0.001) at T1. The DF group showed higher improvements than the standard care in sagittal (Δ−16.49; −27.68 to −5.31), frontal (Δ−13.76; −22.40 to −5.12), and transversal ROM (Δ−15.33; −24.51 to −6.15). The between-group differences were maintained at T2 for all the planes (*p* < 0.001). The DF group showed higher ROM values for sagittal plane (Δ−12.96; −24.50 to −1.42), frontal plane (Δ−11.83; −21.15 to −2.51), and transversal plane (Δ−13.50; −23.32 to −3.67). 

The within-group analysis showed a statistically significant reduction in the standard care in the sagittal ROM at T1 and T2 (*p* < 0.001), as well as in the frontal ROM at T2 (*p* < 0.007) (Table 3). 

### 3.4. Self-Perceived Improvement, Likert Scale

At the T2 follow-up in the standard care, 66.7% felt the same, while 20.5% felt slightly better. In the DF group, 50% felt much better, while 10% very much better. The between-group comparison showed statistically significant results in favor of the DF group (*p* < 0.001).

## 4. Discussion

This randomized controlled clinical trial tried to analyze the effects of three treatment sessions with DF in patients with TTH. Effects on headache frequency, self-perceived impact caused by the headache, cervical ROM, and self-perceived sense of improvement through a Likert scale were assessed. As a treatment protocol, three sessions of 30 min were carried out, and the variables were monitored after the third treatment (T1) and at 1 month follow-up (T2).

TTH is a common symptom with a high prevalence that affects health and causes disability [29,30,31]. The impact that headache has on patients is usually measured through the HIT-6 questionnaire [22]. This study showed that the headache impact decreased in the DF group, while the impact of headache did not vary in the standard care. When analyzing the results between groups, it was observed that instrumental treatment seems to be favorable, with statistically significant results favoring the DF group. However, the change achieved in the DF group (7.82 points) did not reach the minimum clinically significant difference stated in 8 points [22]. Espí-López et al. carried out their research with three treatment groups, the first receiving muscular treatment, the second receiving joint treatment, and the third receiving a combination of both [32]. The third group achieved the greatest change, improving by 7.55 points. In the study conducted by Ferragut-Garcías et al. [33], four groups were included: placebo, muscular treatment, neural treatment, and a combination of the last two. As in the previous study, the group with combined treatment obtained better results, decreasing by 9.4 points in the HIT-6. The HIT-6 reduction was similar in both studies and comparable to our study. However, the treatment dose in both cases, with a total of seven and six treatment days, respectively, was higher than the dosage presented in this study, where three sessions of DF treatment were carried out.

The DF group showed a significant decrease in the number of headache days per month. The standard care showed an increase in frequency without significant results. It is important to consider that the frequency of headache usually fluctuates in patients with chronic TTH or with frequent episodic TTH [34]. It should be noted that both groups followed the standard care treatment and maintained the pharmacological intervention without any modifications in any group. Statistically significant differences were obtained between groups in favor of the DF group (*p* < 0.001; d = 1.12). Headache frequency is usually calculated considering the number of days with headache in the last 2 weeks [21,33]. However, in the present study, the headache frequency per month was recorded. Although variables were not recorded in the same way, the frequency of headache at the beginning of the study (with 13 days per month) seems to be similar to the study of Ferragut-Garcías et al. [33], with 7.2–8 days in 15 days. The data at the end of the study were also similar between both studies, with a decrease in the frequency of headaches. In the trial conducted by Ferragut-Garcías et al. [33] a greater improvement was again observed when treatment techniques were combined. However, the group in which only the soft tissue was treated also improved, as in the present study. 

During this study, the intensity of the headache suffered by the subjects at the time of the evaluation was recorded; this type of recording could explain the low intensity of the headache found. The DF group showed a decrease at T1 and at T2, with statistically significant changes in both. The results did not obtain the minimum clinically important difference stated in 1.3 cm for pain intensities below 3.4 cm [35]. Manual therapy decreased headache intensity in the TTH subgroup [36]. Toro et al. [37] performed two sessions of soft tissue treatment in the suboccipital region, obtaining an immediate improvement in the intensity of the headache. The results of this study, with a lower treatment dose, are in agreement with this study. However, it is not known what occurs at 1 month of follow-up, in which the DF protocol has shown to have positive effects because the intensity of headaches remains practically stable after treatment. 

The limitation of cervical movement Is not typical in patients with TTH, unlike in cervicogenic headache [3]. However, it is an outcome measurement commonly recorded in patients with TTH [36]. It should be noted that for all movements, the intervention group improved the ROM at T1, and the changes were maintained at T2. However, the standard care maintained a restricted ROM or even experienced a reduction in ROM. The main objective of the pharmacological intervention is to relieve symptoms; hence, the lack of changes in cervical ROM may be expected. Other studies with isolated or combined treatments have obtained similar results. Patients seem to improve, although they do not achieve the minimum clinically important difference [21,38,39]. This fact may be related to the lack of ROM restriction or cervical stiffness as an inclusion criterion. Therefore, the patients included in these studies may not present cervical ROM limitations.

The Likert scale allows us to observe how subjects exhibit their sensation. No clinical variable alone can be required for a valid and reliable estimate of clinical changes in neuromusculoskeletal pathologies [40]. For this reason, the patient´s opinion is important for an adequate interpretation of the results, especially in pathologies such as headaches that produce disability and worsening quality of life [41,42,43]. It must be noted that 60% of the DF group felt much better, quite the opposite compared to standard care, where approximately 60% felt practically the same as at the beginning. These results are promising and should be considered for future studies.

Regarding the study’s limitations, it should be noted that, although a standard care was used without changing the medication regimen, it cannot be ruled out if the placebo effect of the applied technique could have caused the results obtained in the DF group. However, since the instrumental placebo treatment suggested in the literature has shown irritating effects for patients, it was decided not to perform it. Secondly, the investigator who performed the treatment technique was not blinded. Thirdly, the examiners registered only the pain related to the TTH, and no other pain conditions that may influence the status of the patients. Fourthly, no selection or comparison by gender was made in our study. Future studies should consider possible differences according to the gender of the patients. Fifthly, there is a lack of knowledge about the adequate treatment dose for TTH. The study suggested three treatments; however, many of the studies with which the results were compared had 6 or more treatment sessions. Lastly, just 1 month follow-up was considered, which could represent a bias due to the follow-up period being too short.

## 5. Conclusions

The addition of three DF treatment sessions in the cervical muscles to a standard pharmacological intervention decreased pain intensity after the intervention; the self-perceived impact caused by the headache, headache frequency, and cervical ROM improved after the intervention and at a 1 month follow-up in TTH patients compared to a standard pharmacological intervention in isolation.

## Figures and Tables

**Table 1:** Sociodemographic and clinical characteristics of each group at T0.

	DF Group (*n* = 40) M (SD)	Standard Care (*n* = 39) M (SD)
Gender (M; F)	11; 29	11; 28
Age (years)	37.25 (15.41)	39.39 (16.26)
Weight (kg)	67.17 (13.95)	67.28 (13.33)
Height (m)	1.67 (0.08)	1.68 (0.06)
BMI (kg/cm^2^)	24.08 (2.17)	23.83 (1.70)
Frequency of headache (days)	13.28 (11.90)	13.26 (12.29)
HIT-6	57.65 (7.51)	56.82 (7.22)
VAS	1.75 (1.74)	2.04 (1.77)
ROM sagittal plane	98.15 (18.65)	106.89 (20.07)
ROM frontal plane	66.97 (14.90)	67.46 (15.49)
ROM transverse plane	115.27 (16.42)	117.23 (17.07)
Pharmacological care		
Acetaminophen	14 (35%)	16 (41%)
NSAIDs	18 (45%)	16 (41%)
Acetaminophen/NSAIDs	8 (20%)	7 (18%)

Abbreviations: M: mean; SD, standard deviation; BMI: body mass index; HIT-6, head impact test; VAS, visual analogue scale; ROM, range of movement. NSAIDs: nonsteroidal anti-inflammatory drugs.

**Table 2:** Differences within and between groups during the study in HIT 6 and the frequency of headaches.

Group	Baseline T0 Mean (SD)	Follow-Up T2 Mean (SD)	Within-Group Score Changes T0–T2 (99% CI)	Between-Group Score Changes
*HIT-6*				
**Standard Care**	56.82 (7.22)	56.49 (7.89)	0.33 (−1.63, 2.30) *p* < 0.649 d = 0.04	F = 27.26 *p* < 0.001 d = 1.17
**DF group**	57.65 (7.51)	49.84 (7.23)	7.82 (4.50, 11.14) *p* < 0.001 d = 1.05	
*Frequency of Headache*				
**Standard Care**	13.26 (12.39)	15.49 (11.98)	−2.23 (−5.06, 0.60) *p* < 0.039 d = 0.18	F = 29.10 *p* < 0.001 d = 1.12
**DF group**	13.28 (11.97)	5.82 (6.99)	7.45 (3.52, 11.37) *p* < 0.001 d = 0.76	

**Abbreviations:** SD: standard deviation; CI: confidence interval; G, group; HIT-6, head impact test.

**Table 3:** Differences within and between groups during the study in VAS and cervical ROM.

Group	Baseline T0 Mean (SD)	Post-Intervention T1 Mean (SD)	Within-Group Score Changes T0–T1 (99% CI)	Between-Group Score Changes T0–T1	Follow-Up T2 Mean (SD)	Within-Group Score Changes T0–T2 (99% CI)	Between-Group Score Changes T0–T2
*VAS*							
**Standard Care**	2.04 (1.77)	2.52 (2.62)	−0.47 (−1.41, 0.47) *p* < 0.181 d = 0.21	F = 11.28 *p* = 0.001 d = 0.76	1.82 (2.08)	0.22 (−0.66, 1.11) *p* < 0.500 d = 0.11	F = 2.36 *p* = 0.129 d = 0.35
**DF group**	1.75 (1.74)	0.74 (1.22)	1.00 (0.26, 1.74) *p* < 0.001 d = 0.67		0.86 (1.55)	0.88 (0.12, 1.63) *p* < 0.003 d = 0.54	
*ROM sagittal plane*							
**Standard Care**	106.90 (20.07)	96.10 (16.67)	10.79 (3.89, 17.69) *p* < 0.001 d = 0.58	F = 39.61 *p* < 0.001 d = 1.42	96.31 (18.37)	10.58 (3.07, 18.10) *p* < 0.001 d =0.55	F= 28.64 *p* < 0.001 d = 1.20
**DF group**	98.15 (18.65)	112.60 (20.69)	−14.45 (−22.80, −6.09) *p* < 0.001 d = 0.73		109.27 (20.37)	−11.12 (−19.13, −3.11) *p* = 0.001 d = 0.57	
*ROM frontal plane*							
**Standard Care**	67.56 (15.49)	65.46 (13.08)	2.00 (−1.20, 5.20) *p* < 0.099 d = 0.15	F = 46.87 *p* < 0.001 d = 1.54	63.54 (14.61)	3.92 (0.20, 7.64) *p* < 0.007 d = 0.27	F= 38.26 *p* < 0.001 d = 1.39
**DF group**	66.97 (14.90)	79.22 (15.83)	−12.25 (−16.85, −7.64) *p* < 0.001 d = 0.79		75.37 (16.64)	−8.40 (−12.30, −4.49) *p* < 0.001 d = 0.40	
*ROM Transverse plane*							
**Standard Care**	117.23 (17.07)	113.59 (15.97)	3.64 (−2.56, 9.84) *p* < 0.120 d = 0.22	F = 27.33 *p* < 0.001 d = 1.17	111.94 (17.69)	5.28 (−0.80, 11.36) *p* < 0.024 d = 0.30	F= 21.86 *p* < 0.001 d = 1.05
**DF group**	115.27 (16.42)	128.92 (13.90)	−13.65 (−20.11, −7.18) *p* < 0.001 d = 0.90		125.45 (15.32)	−10.17 (−16.73, −3.61) *p* < 0.001 d = 0.64	

**Abbreviations:** SD: standard deviation; CI: confidence interval; VAS, visual analogue scale, ROM, range of movement.

## Data Availability

The data analyzed in this study are included in this published article. The dataset is available from the corresponding author on reasonable request.
